# No genetic causal association between circulating alpha-tocopherol levels and osteoarthritis, a two-sample Mendelian randomization analysis

**DOI:** 10.1038/s41598-024-60676-5

**Published:** 2024-05-02

**Authors:** Aiyong Cui, Peilun Xiao, Pengfei Wang, Hu Wang, Yuxuan Cong, Zhiqiang Fan, Xing Wei, Yan Zhuang

**Affiliations:** 1 Department of Orthopaedics, Honghui Hospital, Xi’an Jiao Tong University, Xi’an, 710000 China; 2 Department of Orthopaedics, The Fifth Affiliated Hospital of Sun Yat-Sen University, Zhuhai, 519000 Guangdong China

**Keywords:** Vitamin E, Alpha-tocopherol, Osteoarthritis, Mendelian randomization, Genome-wide association study, Musculoskeletal system, Nutrition

## Abstract

The causal association between vitamin E status and osteoarthritis (OA) remains controversial in previous epidemiological studies. We employed a Mendelian randomization (MR) analysis to explore the causal relationship between circulating alpha-tocopherol levels (main forms of vitamin E in our body) and OA. The instrumental variables (IVs) of circulating alpha-tocopherol levels were obtained from a Genome-wide association study (GWAS) dataset of 7781 individuals of European descent. The outcome of OA was derived from the UK biobank. Two-sample MR analysis was used to estimate the causal relationship between circulating alpha-tocopherol levels and OA. The inverse-variance weighted (IVW) method was the primary analysis in this analysis. We used the MR-Egger method to determine horizontal pleiotropic in this work. The heterogeneity effect of instrumental IVs was detected by MR-Egger and IVW analyses. Sensitivity analysis was performed by removing single nucleotide polymorphism (SNP) one by one. Three SNPs (rs964184, rs2108622, and rs11057830) (*P* < 5E−8) strongly associated with circulating alpha-tocopherol levels were used in this analysis. The IVW-random effect indicated no causal relationship between circulating alpha-tocopherol levels and clinically diagnosed OA (OR = 0.880, 95% CI 0.626, 1.236, *P* = 0.461). Similarly, IVW analysis showed no causal association between circulating alpha-tocopherol levels and self-reported OA (OR = 0.980, 95% CI 0.954, 1.006, *P* = 0.139). Other methods of MR analyses and sensitivity analyses revealed consistent findings. MR-Egger and IVW methods indicated no significant heterogeneity between IVs. The MR–Egger intercept showed no horizontal pleiotropic. The results of this linear Mendelian randomization study indicate no causal association between genetically predicted alpha-tocopherol levels and the progression of OA. Alpha-tocopherol may not provide beneficial and more favorable outcomes for the progression of OA. Further MR analysis based on updated GWASs with more IVs is required to verify the results of our study.

## Introduction

Osteoarthritis (OA) is a chronic, progressive degenerative joint disease, mainly presented by the deterioration of articular cartilage and leading to high disability in daily activities and reduced life quality^1^. It is estimated that over 500 million individuals are currently suffering from OA worldwide, and one-third of the population over 65 years old is affected by OA. As a result of the aging population and the obesity epidemic, the prevalence of OA has been increasing in recent years, placing a significant health and economic burden on society^2^. Since there is no effective cure for OA except total joint replacement, the treatment strategy for osteoarthritis has shifted to prevention at an early stage.

Vitamin E is a lipid-soluble vitamin that has been proven to be a powerful antioxidant in our bodies. Vitamin E consists of eight isoforms, including four tocotrienols (α, β, γ, δ) and four tocopherols (α, β, γ, δ). Alpha-tocopherol and gamma-tocopherol are two primary forms in the body^3^, which play some fundamental physiological roles through their potent antioxidant properties^4,5^. Vitamin E status has been proven to be associated with many diseases, including cancers, cardiovascular disease, renal disease, and dementia^6–9^. The association between vitamin E and OA has also been widely discussed. Oxidative stress is an essential mechanism that has been mentioned in the occurrence of OA. Recently, an increasing number of studies have demonstrated the possible role of vitamin E in the development of OA because of its anti-inflammatory and antioxidant effects^10–12^. In the animal experiment, vitamin E showed the ability to protect rat mesenchymal stem cells (MSCs) from hydrogen peroxide-induced oxidative stress^12^. Then, they implanted vitamin E pretreated MSCs into the OA rat model and found that the progression of OA had been impeded. However, clinical studies exploring the relationship between vitamin E and OA have always shown contradictory results. In a study in north India, Bhattacharya et al.^10^ found that inflammatory markers like erythrocyte malondialdehyde level and antioxidant enzymes have significantly reduced after three months of vitamin E treatment in OA patients. In an early prospective study, McAlindon et al.^13^ revealed that higher vitamin E intake could reduce the risk of OA progression after adjusting a range of confounders. However, a cross-sectional study of 4685 participants revealed that dietary vitamin E was not significantly related to radiographic knee OA^14^. In another random double-blind study, Brand et al.^15^ found that six months of 500 IU/day vitamin E supplement could not relieve the knee OA symptom compared with the placebo group.

These inconsistent results may be attributed to different study populations, designs, and inevitable confounders in clinical studies. Mendelian randomization (MR) is a novel method using a genetic variation to assess the causal relationship between exposure and outcome^16^. It could avoid confounding factors and infer causality since the alleles of exposure genetic variants are randomly assigned^17^. Circulating alpha-tocopherol levels have been used to predict various diseases at the genetic level, such as Alzheimer’s, cardiovascular disease, and cancers^6,18,19^. However, no study has assessed the genetic association between circulating alpha-tocopherol levels and OA. Thus, we employed an MR analysis using genetic variations to evaluate the causal relationship between circulating alpha-tocopherol levels and OA.

## Materials and methods

### Study design

**Figure 1:**
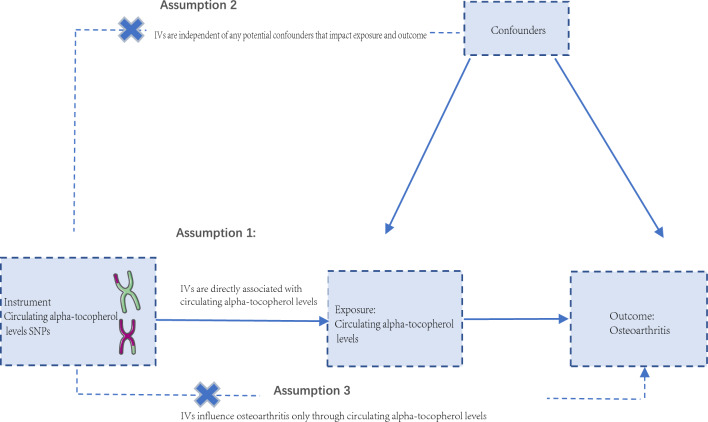
The study design of two‐sample MR analysis.

The MR analyses conform to three assumptions: 1. Selected single nucleotide polymorphism (SNP) should be strongly correlated with exposure. 2. Selected SNPs are not related to the outcome through confounders. 3. Selected SNPs are supposed to affect outcomes via exposure, but not the direct association (Fig. 1).

### Data sources for alpha-tocopherol levels

From a GWAS including 7,781 individuals of European descent, three SNPs (rs964184, rs2108622, and rs11057830) (*P* < 5E−8) strongly associated with circulating alpha-tocopherol levels were identified. The GWAS analysis was adjusted for age, body mass index (BMI), cancer status, and cholesterol^20^. These IVs closely associated with circulating alpha-tocopherol levels have been widely used in previous MR studies^6,18,19^. Studies showed that higher circulating alpha-tocopherol level was genetically associated with increased risk of coronary artery disease, myocardial infarction, and bladder cancer but decreased risk of breast cancer^6,19^. First, the SNPs of circulating alpha-tocopherol were concluded in the Alpha-Tocopherol, Beta-Carotene Cancer Prevention Study cohort (ATBC) (n = 4014). Then, the authors replicated the above results in a meta-analysis of the Prostate, Lung, Colorectal, and Ovarian Cancer Screening Trial (PLCO) study (n = 992) and the Nurses’ Health Study (NHS) (n = 2775)^20^. Of the 7781 individuals of European descent included in this GWAS, 48% were American, and 52% were residents of southwestern Finland, with a mean age of 60.6 ± 5.4 years. Details on SNPs for alpha-tocopherol levels were summarized in Table 1. Table S1 summarized the participants’ inclusion and exclusion criteria for the GWAS that identified SNPs of circulating alpha-tocopherol levels.

**Table 1:** Characteristics of SNPs associated with circulating alpha-tocopherol levels.

SNP	Nearby Gene	Chromosome	Sample size	EA	OA	EAF	SNP-exposure (Alpha-tocopherol levels)			SNP-outcome (Osteoarthritis)		
							Beta	SE	*P*	Beta	SE	*P*
Alpha-tocopherol levels												
rs11057830	12	SCARB1	7781	A	G	0.15	0.03	0.01	8.20E−09	0.0045	0.0110	0.682
rs2108622	19	CYP4F2	7781	T	C	0.21	0.03	0.01	1.40E−10	− 0.004	0.0083	0.640
rs964184	11	BUD13/ZNF259/APOA5	7781	G	C	0.15	0.04	0.01	7.80E−12	− 0.012	0.0112	0.307

*P* value < 5 × 10^–8^ for reporting genome-wide significance.

*EA* effect allele, *OA* other allele, *EAF* effect allele frequency, *MR* Mendelian randomization, *SE* standard error, *SNP* single nucleotide polymorphism.

### Data sources for osteoarthritis

The primary outcome was the clinically diagnosed OA, which was obtained from a large published GWAS^21^. The GWAS was conducted in 403,124 European individuals for K/HOA (24,955 patients with KOA and 378,169 controls). The OA was identified depending on clinical evidence of a disease needing joint arthroplasty or imaging evidence that Kellgren-Lawrence grade ≥ 2). The second outcome of OA (self-reported OA at any site) was derived from the UK Biobank (UK Biobank: 20002#1465), with a total of 38,472 OA patients and 424,461 control samples. The GWAS of OA in different genders was also derived from the UK biobank. The detailed source of GWAS datasets can be found in Table S2. Table S3 showed the STROBE-MR checklist.

### Statistical analysis

The data were harmonized in exposure and outcome data to ensure allelic concordance. The MR approach was conducted to estimate the causality between circulating alpha-tocopherol levels and OA. Five methods were employed to examine the causal association between circulating alpha-tocopherol levels and OA, including inverse-variance weighted (IVW), MR-Egger, weighted mode, weighted median, and simple model, among which IVW was the primary method^22^. Random-effects IVW was performed to assess the genetic predictions between circulating alpha-tocopherol levels and OA. We also performed subgroup analyses of different genders. The MR-Egger method was used to test its horizontal pleiotropic. MR-PRESSO global test could not be used due to limited IVs. Cochran’s Q statistic was applied to examine the heterogeneity of individual SNPs in IVW and MR-Egger tests. We additionally performed sensitivity analysis by removing single SNP one by one. To evaluate the weak instrument bias, we calculated the F statistics using the formula ${\text{F}}=(\frac{N-K-1}{K})(\frac{{R}^{2}}{1-{R}^{2}})$, where N is the sample size, K is the number of IVs, and *R*^2^ is the proportion of the variability of the exposure explained by IVs. All data were analyzed by package “TwoSampleMR” of the R language.

### Ethical approval

This research doesn’t need any ethics approval.

## Results

### Power analysis

Three SNPs (rs964184, rs2108622, and rs11057830) (*P* < 5E−8) strongly associated with circulating alpha-tocopherol levels were used in this analysis. The proportion of alpha-tocopherol variance (R^2^) explained by these three genetic variants is estimated at 1.15%. The F-statistic for IVs (including these 3 IVS) was 247.83 > 10, indicating a weak bias of the IVs (Table 1).

### The causal association between circulating alpha-tocopherol levels and clinically diagnosed OA

**Figure 2:**
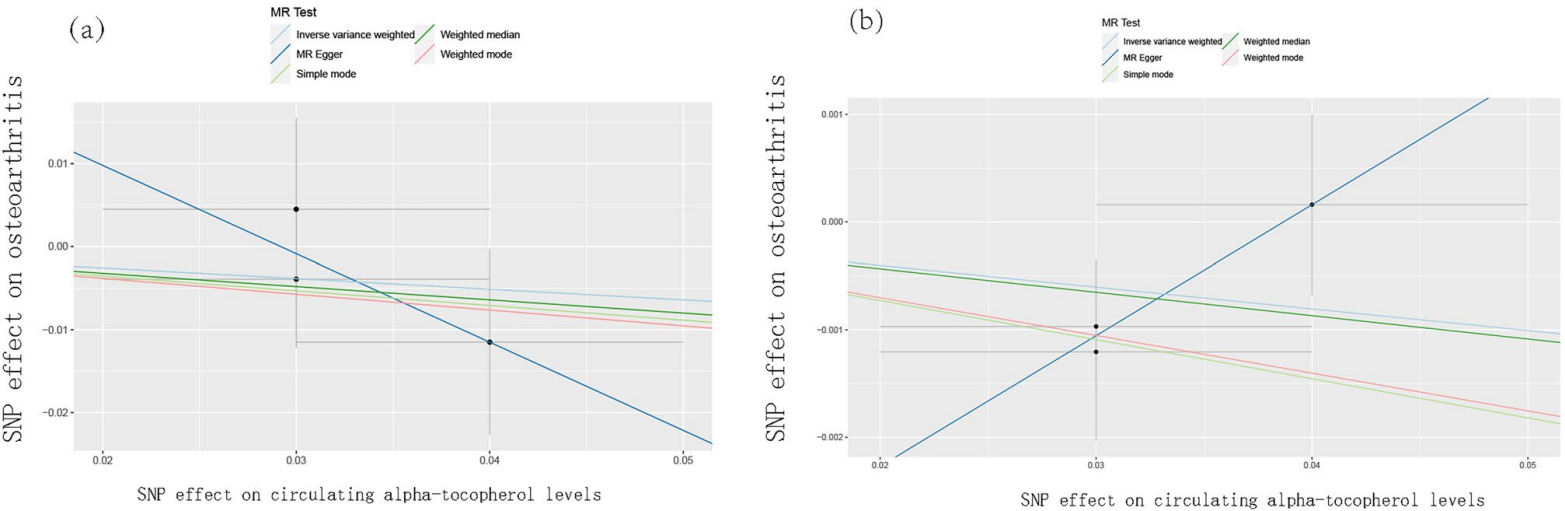
The scatter plot for MR analyses. (**a**): circulating alpha-tocopherol levels and clinically diagnosed OA. (**b**) circulating alpha-tocopherol levels with self-reported OA. Abbreviations: IVW: inverse-variance weighted. OA: osteoarthritis.

**Figure 3:**
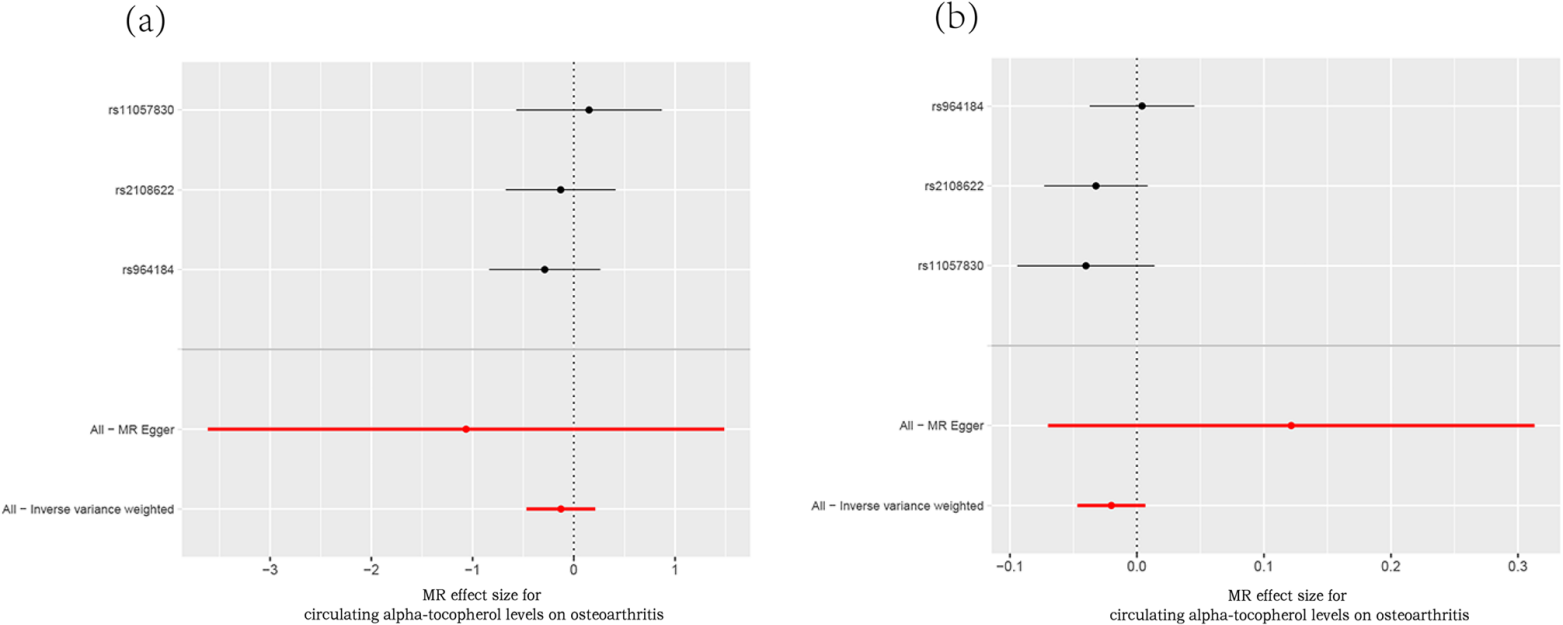
The forest plot for MR analyses. (**a**): circulating alpha-tocopherol levels and clinically diagnosed OA. (**b**) circulating alpha-tocopherol levels with self-reported OA.

**Figure 4:**
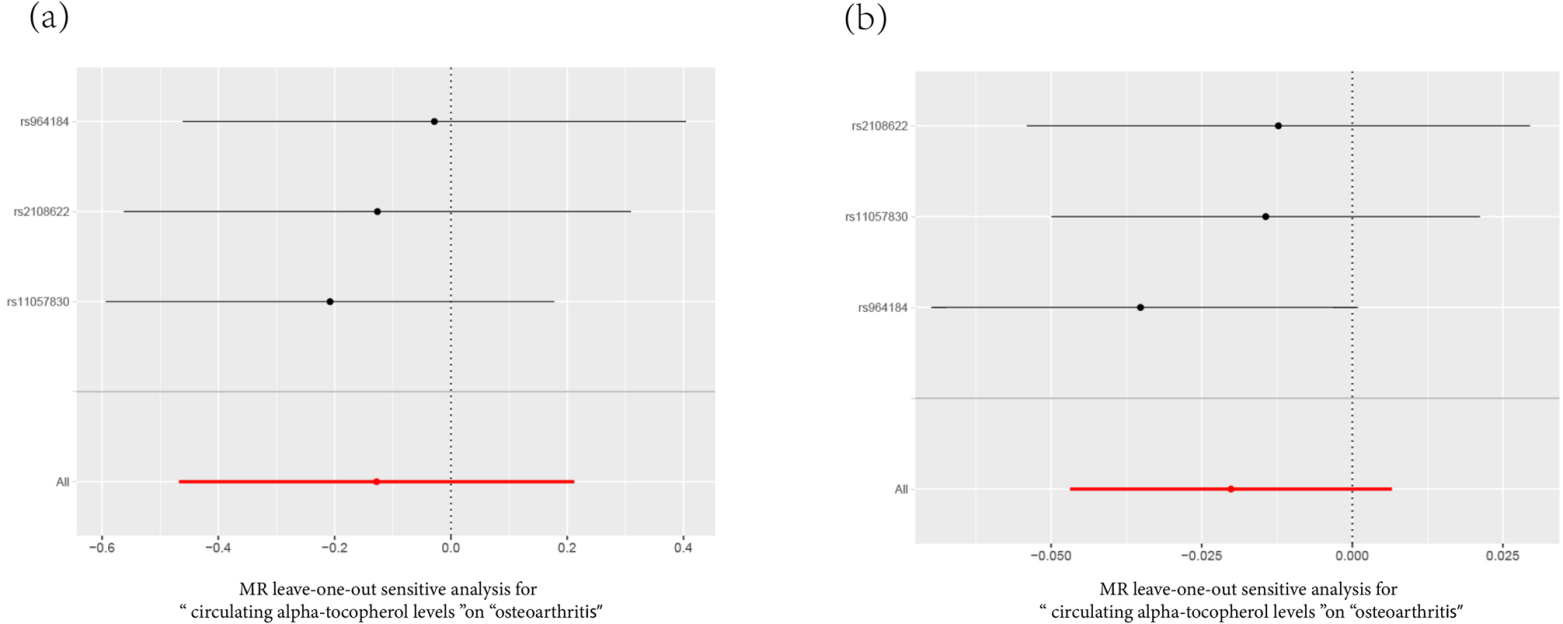
Leave-one-out sensitivity analysis for MR analysis. (**a**): circulating alpha-tocopherol levels and clinically diagnosed OA. (**b**) circulating alpha-tocopherol levels with self-reported OA.

The causal association of alpha-tocopherol variants with clinically diagnosed OA was shown in Fig. 2a and Table 2. The IVW results found no causal relationship between circulating alpha-tocopherol levels and clinically diagnosed OA (OR = 0.880, 95% CI 0.626, 1.236, *P* = 0.461). Similarly, no causal association was observed in either the weighted median, MR-Egger test, weighted mode, or simple mode. The Cochran’s Q statistic of MR-Egger (*P* = 0.542) and IVW methods (*P* = 0.638) indicated no significant heterogeneity between IVs. The MR–Egger intercept in the analysis showed no horizontal pleiotropic (*P* = 0.600). The forest plots were shown in Fig. 3a. The results were also consistent after the sensitivity analysis (Fig. 4a).

**Table 2:** Causal effect of circulating alpha-tocopherol levels with OA.

Exposure	Outcome	No. of SNP	Method	OR (95% CI)	*P* for Association	*P* for Heterogeneity Test	*P* for MR-Egger Intercept	Statistical Power
Alpha-tocopherol	Clinically diagnosed OA	3	IVW	0.880 (0.626, 1.236)	0.461	0.638	0.600	
								247.83
		3	MR Egger	0.345 (0.027, 4.418)	0.563	0.542		
		3	Weighted median	0.852 (0.387, 1.875)	0.493			
		3	Simple mode	0.838 (0.520, 1.351)	0.543			
		3	Weighted mode	0.827 (0.507, 1.346)	0.524			
Alpha-tocopherol	Self-reported OA	3	IVW	0.980 (0.954, 1.006)	0.139	0.334	0.382	
		3	MR Egger	1.129 (0.932, 1.368)	0.431	0.819		
		3	Weighted median	0.979 (0.946, 1.012)	0.203			
		3	Simple mode	0.964 (0.922, 1.008)	0.251			
		3	Weighted mode	0.966 (0.919, 1.015)	0.300			

*CI* confidence interval, *MR* Mendelian randomization, *IVW* inverse-variance weighted, *SNP* single nucleotide polymorphism, *OA* osteoarthritis.

### The causal association between circulating alpha-tocopherol levels with self-reported OA

The causal association of alpha-tocopherol variants with self-reported OA also showed consistent findings, which can be found in Fig. 2b and Table 2. The IVW results found no causal relationship between circulating alpha-tocopherol levels and self-reported OA (OR = 0.980, 95% CI 0.954, 1.006, P = 0.139). No causal association was also found in either the weighted median, weighted mode, MR-Egger test, or simple mode. The Cochran’s Q statistic of MR-Egger (*P* = 0.819) and IVW methods (*P* = 0.334) indicated no significant heterogeneity between IVs. The MR–Egger intercept in the analysis showed no horizontal pleiotropic (*P* = 0.382). The forest plots were shown in Fig. 3b. The results were also consistent after the sensitivity analysis (Fig. 4b).

### The causal association between circulating alpha-tocopherol levels with self-reported OA by gender

We conducted the subgroup analysis by gender (Table 3). The IVW results showed no causal relationship between circulating alpha-tocopherol levels and self-reported OA in men (OR = 0.983, 95% CI: 0.927, 1.042, P = 0.562) and women (OR = 0.967, 95% CI: 0.922, 1.013, P = 0.156). The Cochran’s Q statistic of MR-Egger (P = 0.080 for men; P = 0.353 for women) and IVW methods (P = 0.090 for men; P = 0.293 for women) indicated no significant heterogeneity between IVs. The MR–Egger intercept in the analysis showed no horizontal pleiotropic (P = 0.590 for men; P = 0.427 for women).

**Table3:** The causal association between circulating alpha-tocopherol levels with OA by gender.

Gender	No. of SNP	Method	OR (95% CI)	*P* for Association	*P* for Heterogeneity Test	*P* for MR-Egger Intercept
Men	3	IVW	0.983 (0.927–1.042)	0.562	0.090	0.590
	3	MR Egger	1.185 (0.723–1.945)	0.622	0.080	
	3	Weighted median	1.001 (0.943–1.062)	0.972		
	3	Simple mode	1.005 (0.950–1.062)	0.885		
	3	Weighted mode	1.005 (0.954–1.058)	0.875		
Women	3	IVW	0.967 (0.922–1.013)	0.156	0.293	0.427
	3	MR Egger	0.789 (0.574–1.084)	0.382	0.353	
	3	Weighted median	0.964 (0.906–1.026)	0.249		
	3	Simple mode	0.960 (0.894–1.030)	0.371		
	3	Weighted mode	0.959 (0.897–1.025)	0.340		

*CI* confidence interval, *MR* Mendelian randomization, *IVW* inverse-variance weighted, *SNP* single nucleotide polymorphism, *OA* osteoarthritis.

## Discussion

In this genetic association study, we evaluated the causal association between alpha-tocopherol levels and OA risk based on 7,781 European descent individuals and the large UK Biobank individual-level datasets. Our two-sample MR analysis did not support a causal association between circulating alpha-tocopherol levels and OA in individuals of European descent. The association was also observed in both men and women. Alpha-tocopherol levels may not provide beneficial and more favorable outcomes for the progression of OA.

Oxidative stress is one of the essential mechanisms in the pathogenesis of OA^23^. Evidence showed that increased oxidative stress could be harmful to the chondrocytes by activating the path like c-Jun N-terminal kinase (JNK) and phosphoinositide 3-kinase (PI3K), and then lead to osteoarthritis^24^. In previous studies, vitamin E or alpha-tocopherol levels has been widely discussed as a possible agent for the prevention and treatment of OA because of its anti-inflammatory and antioxidant properties^11,25^. Some vitro experiments observed a positive effect of vitamin E or alpha-tocopherol levels on preventing the progression of OA^26–28^. Tiku et al.^26^ found that vitamin E could prevent cartilage matrix degradation induced by hydrogen peroxide and calcium ionophores. They also observed that vitamin E reduces oxidative stress markers in human chondrocytes like hydroxynonenal protein and malondialdehyde. Beecher et al.^28^ investigated the effect of several antioxidants on mechanical stress-induced cartilage damage and found that vitamin E slowed down chondrocyte apoptosis induced by mechanical stress. Several animal experiments also suggested that vitamin E can prevent joint degeneration and improve animal function^12,29^. Rhouma et al.^29^ explored the effects of vitamin E on dogs. They found that dogs in the vitamin E supplement group had lower pain visual analog scores and cartilage damage scores after manual removal of the cruciate ligament to induce osteoarthritis. In a recent study, Bhatti et al.^12^ found that vitamin E pretreatment enables mesenchymal stem cells to counteract H_2_O_2_-induced oxidative stress and increases cartilage matrix proteoglycan content in mice. Another study by Ozkan et al. showed that vitamin E treatment improved rat joint histological scores^30^. Nevertheless, the causal relationship association between vitamin E status and OA remains controversial in epidemiological studies. In a Japanese survey of 827 rural participants, the researcher found a negative association between vitamin E intakes and knee osteoarthritis after adjusting age, total energy, and body mass index in females but not males^31^. However, vitamin E intakes in this study was calculated by diet history questionnaire, which could be influenced by individuals’ recall bias. A cross-sectional study of 562 individuals showed that individuals with the middle tertile of serum alpha-tocopherol have the lowest OA risk, which may imply a U-shaped between vitamin E status and OA^32^. In another study, 3,026 individuals aged 50 to 79 years were enrolled, Chaganti et al.^33^ found that the highest tertile of serum alpha-tocopherol level had a higher risk of OA than the lowest tertile (OR = 1.89, 95CI 1.02–3.50, *P* = 0.042) after logistic regression.

Consistent with our study, some clinical studies have shown no association between vitamin E status and OA risk^14^. In a study with a large sample size (4,685 Chinese), Li et al. found no relationship between dietary intakes of vitamin E and radiographic knee OA after adjusting a large range of potential confounding factors^14^. In another randomized controlled trial by Aydogan et al. ^34^, compared with the controlled group, oral vitamin E combined with intra-articular Hylan G-F 20 treatment did not significantly improve oxidative stress markers in the blood and synovial membranes of patients with OA. Another 2-year, double-blind, randomized, placebo-controlled study came to the same conclusion^35^. In this study, 136 patients with knee OA were randomly divided into the experiment group (receiving vitamin E (500 IU) for two years) or the control group (receiving a placebo for two years). The authors found no symptom improvement in the vitamin E treatment group compared with the placebo treatment group^35^. These epidemiological studies are prone to generate biased results because of the residual confounding and reverse causation^36^. At the genetic level, SNPs of circulating alpha-tocopherol have been used to predict various diseases, such as Alzheimer’s, cardiovascular disease, and cancers^6,18,19^. We are the first MR study to demonstrate that alpha-tocopherol is not associated with the progression of OA.

Our study has several advantages. First, MR analysis was designed to reduce confounding and reverse causality using genetic variation, as SNPs are randomly assigned at conception. Second, we used a large sample of alpha-tocopherol GWAS dataset (7781 Europeans) to conduct this MR analysis. Two large OA GWAS datasets (clinically diagnosed OA and self-reported OA) were used for replicate analyses, which improved the credibility of our study. Third, the results of the five methods of MR analysis were consistent, which added to the evidence of our study. Fourth, the overall heterogeneity was low, verified by MR-Egger and IVW methods. No horizontal pleiotropic was found by MR-Egger regression. The results of the sensitive analysis were consistent after omitting single SNP one by one. Fifth, we conducted subgroup analyses by gender and obtained similar results, further supporting our conclusions.

However, some potential limitations could not be avoided. First, alpha-tocopherol and gamma-tocopherol are two primary forms in the body. In our MR analysis, we selected only alpha-tocopherol levels to represent circulating vitamin E levels. Previous studies suggested γ-tocopherol may play a unique role apart from alpha-tocopherol in our bodies^37^. Thus, further studies with more robust evidence should investigate the effect of different vitamin E isoforms on OA. Second, the number of SNPs related to circulating alpha-tocopherol levels is limited (only 3 SNPs). It could impact pleiotropy detection by MR Egger regression, although the results showed no horizontal pleiotropic. Further MR analysis based on updated GWASs with more IVs is required to verify the results of our research. Third, we used a linear MR analysis to explore a causal relationship between circulating alpha-tocopherol and OA. However, there may be a threshold effect for the alpha-tocopherol on OA, and nonlinear MR analysis will be needed in the future to validate our conclusions. Fourth, these findings should be interpreted cautiously, as the study sample was from a European population. Whether the results can be generalized to other populations needs further investigation.

## Conclusions

The results of this linear Mendelian randomization study indicate no causal association between genetically predicted circulating alpha-tocopherol levels and the progression of OA. Alpha-tocopherol may not provide beneficial and more favorable outcomes for the progression of OA. Further MR analysis based on updated GWASs with more IVs is required to verify the results of our study.

### Supplementary Information

Supplementary Table S1.

Supplementary Table S2.

Supplementary Table S3.

## Data Availability

The data used in this study could be found at GWAS studies we mentioned and UK biobank (https://www.ukbiobank.ac.uk/).
