# Supplementary material for: No genetic causal association between circulating alpha-tocopherol levels and osteoarthritis, a two-sample Mendelian randomization analysis
Source: Sci Rep. 2024 May 2;14:10099. doi: 10.1038/s41598-024-60676-5 (PMC11066079; doi:10.1038/s41598-024-60676-5)
Supplement: Supplementary file 1 — Supplementary Table S1. [file 41598_2024_60676_MOESM1_ESM.pdf]

Table S1: Inclusion and exclusion criteria for the genome-wide association study that identified SNPs of circulating alpha-tocopherol levels.

	Inclusion criteria	Exclusion criteria
**Trial cohort**		
ATBC Study	Male smokers at study entry, aged 50– 69 years, and residents of southwestern Finland.	History of cancer, had severe diseases limiting long-term participation or took supplements of vitamins E (>20 mg/day) or A (>20 000 IU/day) or beta-carotene (>6 mg/day)
**Replicated cohort**		
PLCO Study	Male and female participants of Caucasian descent, aged 55 to 74 years.	-
NHS Study	Female registered nurses, aged 30 to 55 years.	-

Abbreviations: ATBC, Alpha-Tocopherol, Beta-Carotene Cancer Prevention Study cohort; PLCO, Prostate, Lung, Colorectal, and Ovarian Cancer Screening Trial; NHS, Nurses' Health Study; SNPs, single nucleotide polymorphisms.
