# Supplementary material for: No genetic causal association between circulating alpha-tocopherol levels and osteoarthritis, a two-sample Mendelian randomization analysis
Source: Sci Rep. 2024 May 2;14:10099. doi: 10.1038/s41598-024-60676-5 (PMC11066079; doi:10.1038/s41598-024-60676-5)
Supplement: Supplementary file 2 — Supplementary Table S2. [file 41598_2024_60676_MOESM2_ESM.pdf]

Supplementary table 2: A detailed description of the GWAS data involved in this study.

Trait	Study/Consortium	Ancestry	Sample size	Data available
alpha-tocopherol levels	Study by Major et al.	European	7,781	https://doi.org/10.1093/hmg/ddr296
Clinically diagnosed OA	Study by Tachmazidou et al.	European	403,124	https://www.nature.com/articles/s41588-018-0327-1
Self-reported OA	UK biobank	European	462,933	https://broad-ukb-sumstats-us-east-1.s3.amazonaws.com/round2/additive-tsvs/20002_1465.gwas.imputed_v3.both_sexes.tsv.bgz
Self-reported OA (men)	UK biobank	European	166,988	https://broad-ukb-sumstats-us-east-1.s3.amazonaws.com/round2/additive-tsvs/20002_1465.gwas.imputed_v3.female.tsv.bgz
Self-reported OA (women)	UK biobank	European	194,153	https://broad-ukb-sumstats-us-east-1.s3.amazonaws.com/round2/additive-tsvs/20002_1465.gwas.imputed_v3.male.tsv.bgz
